# Airway and Ventilation Rescue During Tracheal Resection: A Case Report of Intraoperative Troubleshooting

**DOI:** 10.7759/cureus.107007

**Published:** 2026-04-14

**Authors:** Taha B Aljishi, Sylvain Gagné, Calvin Thompson, Ryan McGinn, Donna Maziak

**Affiliations:** 1 Department of Anesthesiology, King Fahad Specialist Hospital, Dammam, SAU; 2 Department of Anesthesiology and Pain Medicine, The Ottawa Hospital, University of Ottawa, Ottawa, CAN; 3 Department of Surgery, The Ottawa Hospital, University of Ottawa, Ottawa, CAN

**Keywords:** bronchial blocker, cross-field ventilation, high-frequency jet ventilation, jet ventilation, lung isolation, tracheal resection, tracheal tumor

## Abstract

Tracheal resection requires shared airway management when conventional ventilation fails. The present paper describes a 73-year-old man with chronic obstructive pulmonary disease (COPD), hypertension, and prior neck radiotherapy who underwent tracheal resection with primary anastomosis for chondrosarcoma. Initial intubation with a single-lumen endobronchial tube produced a major leak, requiring exchange to an endotracheal tube. Lung isolation with an EZ blocker® (Teleflex Life Sciences Ltd., Athlone, Ireland) was achieved but lost during thoracotomy in the lateral decubitus position with a chin stitch; an Arndt blocker also failed. Ventilation transitioned to high-frequency jet ventilation with intermittent apnea, cross-field ventilation, and intermittent endotracheal tube ventilation. A cuff rupture causing a leak was managed with cuff inflation, allowing successful extubation. The patient remained hemodynamically stable without complications and was discharged one week postoperatively at his functional baseline, with adequate oxygenation, good pain control, and full independence.

## Introduction

Tracheal tumor resection remains one of the most demanding scenarios in thoracic anesthesia. These cases often involve central airway obstruction, and the airway must be shared between anesthesiologists and surgeons. Loss of effective oxygenation or ventilation can be catastrophic; therefore, airway management must be individualized with clear primary and rescue pathways [1,2]. Few operative procedures rely as heavily on comprehensive preoperative planning and well-coordinated interdisciplinary collaboration to ensure safety and success [2,3].

Multiple airway techniques have been described for tracheal resection to maintain oxygenation and ventilation while preserving surgical exposure, including bypassing the lesion with an endotracheal tube, rigid bronchoscopy with endoluminal debulking, laser therapy and stenting, high-frequency jet ventilation (HFJV), and cardiopulmonary bypass or extracorporeal support in select cases. Choice of technique depends on the surgical approach and local expertise [1,4]. Because airway conditions may change intraoperatively, the literature emphasizes careful preoperative lesion characterization, explicit surgeon-anesthesiologist communication, and readily deployable backup strategies if the initial plan fails.

In this report, we describe anesthetic management for tracheal resection in a patient who had predictors of a difficult airway, with airway manipulation further constrained by decubitus positioning and a chin-to-chest suture. We were unable to maintain lung isolation despite sequential bronchial blocker techniques, necessitating escalation to jet ventilation with intermittent apnea and cross-field ventilation - a technique in which a sterile endotracheal or endobronchial tube is placed directly into the distal airway across the open surgical field to maintain ventilation during airway reconstruction when conventional intubation is not feasible - during which clinically relevant limitations were encountered. The case was further complicated by endotracheal cuff rupture requiring continuous cuff inflation to mitigate a major leak, ultimately allowing safe completion of surgery.

This case illustrates the dynamic challenges of airway and ventilation management during tracheal resection and highlights practical troubleshooting strategies when lung isolation techniques fail. The patient provided a written consent for hospital use of clinical data, images, and information for educational and quality-improvement purposes with the understanding that confidentiality would be protected. This case report contains no patient-identifying information and no personal photographs.

## Case presentation

A 73-year-old man (BMI 27.4) with chronic obstructive pulmonary disease (COPD), hypertension (HTN), and recent head-and-neck radiotherapy for tonsillar squamous cell carcinoma (SCC) was scheduled for elective tracheal resection with primary anastomosis for an incidentally discovered chondrosarcoma. He reported only a chronic productive cough. Airway exam suggested difficulty (Mallampati III, limited mouth opening and neck range of motion).

**Figure 1:**
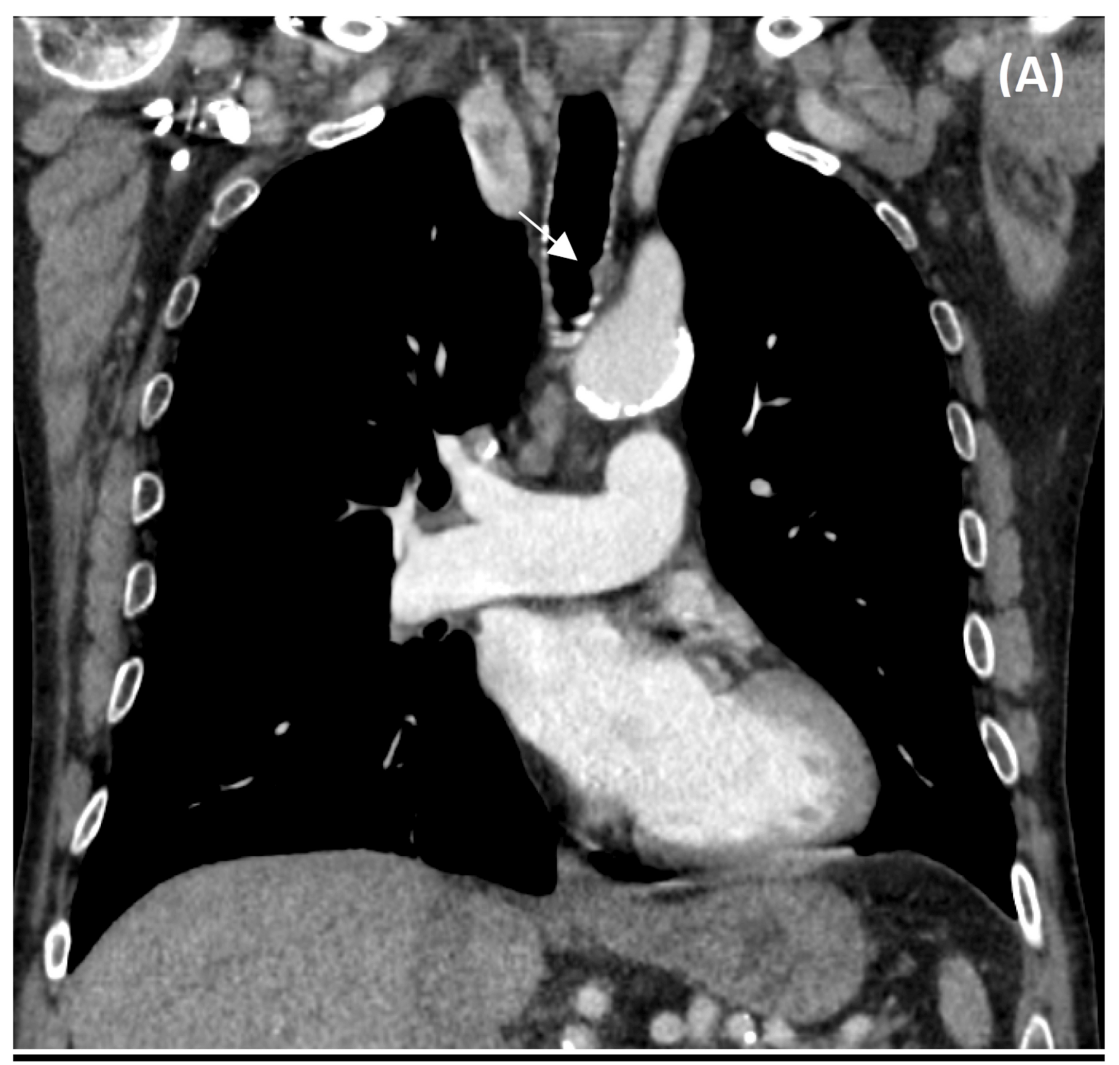
Well-circumscribed soft tissue nodule in the anterior aspect of the trachea, approximately 8 cm above the carina, disrupting the cartilaginous wall with small extension to the lumen.

Preoperative computerized tomography and bronchoscopy demonstrated an anterior tracheal lesion (~1.5 cm) ~7 cm from the glottis and ~8 cm from the carina with <25% luminal obstruction (Figure 1).

Preoperative biopsy confirmed low-grade chondrosarcoma. A preoperative multidisciplinary team, including anesthesiologists and surgeons, coordinated a comprehensive airway and surgical plan, involving initial use of a 7.5 mm armored single-lumen endobronchial tube with the ability to facilitate subsequent lung isolation and allow bronchial blocker placement if required, progression from cervical mediastinoscopy to right thoracotomy with planned left mainstem intubation for lung isolation during tracheal resection and anastomosis, and predefined backup strategies including high-frequency jet ventilation and cross-field ventilation.

We placed a left paramedian thoracic epidural (T4-5), large-bore peripheral intravenous access (16G catheter in the left hand and 18G catheters in the right hand and right foot), and an arterial line before induction. Anesthesia was induced and maintained with total intravenous anesthesia (TIVA) using infusions of propofol, with depth of anesthesia titrated based on SedLine® waveform monitoring (Masimo, Irvine, USA). We selected a 7.5 mm armored single-lumen endobronchial tube, which is usually advanced into a mainstem bronchus and has a cuff positioned within the bronchus, typically smaller and designed to seal a narrower lumen, for initial airway management, given the planned sequence of surgery - cervical mediastinoscopy without immediate need for lung isolation - with the added advantage that it could later facilitate lung isolation during thoracotomy, allow intraoperative manipulation of tube position, and permit placement of a backup bronchial blocker through the lumen if required. Initial video laryngoscopy (VL) endotracheal intubation attempt with an endobronchial tube was complicated by a persistent cuff leak when the tube was positioned in the trachea despite maximal cuff inflation per the manufacturer's instructions; this was attributed to the patient's large tracheal diameter, with no larger single-lumen endobronchial tube available. We exchanged the tube for a 7.5 mm armored endotracheal tube (ETT). We did not choose a double-lumen tube for initial airway management due to its larger caliber, which carries an increased risk of airway trauma, edema, and postoperative complications; this was particularly relevant in our case, where repeated proximal and distal manipulation of the tube relative to the lesion was planned initially, further increasing the risk of airway injury. The procedure began with a cervical mediastinoscopy with two-lung ventilation. We placed an EZ-Blocker® (Teleflex Life Sciences Ltd., Athlone, Ireland) under fiberoptic guidance (FO), and we achieved right lung isolation. The case then proceeded to a right thoracotomy with the patient positioned in lateral decubitus, with a chin-to-chest suture.

During thoracotomy and during surgical exposure, the surgical team noted loss of lung isolation. At this stage, oxygenation and ventilation were preserved, allowing time to determine the next management steps. There was continuous communication between the anesthesia and surgical teams to consider workable alternatives to restore lung isolation. Exchange from an EZ-Blocker to an Arndt blocker failed to restore isolation.

The case proceeded with high-frequency jet ventilation (HFJV) through the endotracheal tube, aiming for an endobronchial jet insufflation and lung isolation, which was complicated by frequent high-pressure alarms, continued loss of lung isolation, and requiring intermittent apnea (ventilation resumed when peripheral oxygen saturation (SpO₂) fell below 88%) and, when feasible, cross-field ventilation. During resection, high-frequency jet ventilation (HFJV) was limited by the resected tissue debris, blood, and secretions contaminating both lungs, requiring frequent bronchial toileting.

During the procedure, the ETT was repositioned under FO guidance, according to the surgeon's instructions, proximal or distal to the lesion. During these adjustments, we identified a significant leak and attributed it to a ruptured ETT cuff. As a rescue measure, we maintained continuous cuff inflation using an auxiliary oxygen line from the anesthesia machine, which compensated for the leak and allowed completion of the mass resection with primary anastomosis without complication. The sequential airway management strategies and intraoperative challenges are summarized in Table 1.

**Table 1:** Sequential airway management strategies and intraoperative troubleshooting during tracheal resection. EZ blocker® (Teleflex Life Sciences Ltd., Athlone, Ireland)

Phase	Airway Device	Lung Isolation	Limitation	Intervention
Induction	Endobronchial tube (ETT)	Not required	Cuff leak	Exchanged for an ETT
Mediastinoscopy	Endotracheal tube	Not required	None	Proceeded without change
Thoracotomy	EZ-Blocker®	Achieved	Blocker malposition	Replaced with an Arndt blocker
Thoracotomy	Arndt blocker	Lost	Blocker malposition	Transitioned to high-frequency jet ventilation (HFJV)
Thoracotomy and Resection	HFJV	Limited	High-pressure alarms, blood, and debris contaminating the airway	Bronchial toileting, intermittent apnea, cross-field ventilation
Reconstruction	ETT with intermittent apnea, cross-field ventilation, and HFJV	Limited	Endotracheal cuff rupture	Continuous cuff inflation

At the end of the procedure, we extubated the patient in a semi-sitting position on a low-dose remifentanil and dexmedetomidine infusion to minimize coughing, while maintaining neck flexion with a chin stitch. He was then connected to high-flow nasal cannula (HFNC) therapy and transferred to a thoracic observation unit.

The patient remained hemodynamically stable throughout his hospital stay without any signs of end-organ injury. By the time of discharge - one week after the operation - he was maintaining oxygen saturations, tolerating a regular diet, fully ambulatory at his independent baseline, and his pain was well controlled.

## Discussion

Tracheal resection is a high-risk shared-airway procedure in which the anesthetic priority of reliable oxygenation and ventilation must be balanced with the surgical need for unobstructed access during resection. No single airway or ventilation technique is universally superior; strategies must be individualized to obstruction severity, lesion size and location, surgical approach, comorbidities, team expertise, and available resources. Of notable importance, explicit primary plans should be supported by readily deployable rescue pathways [1,2]. This case illustrates how even an initially workable strategy can become nonfunctional as airway geometry changes with surgical manipulation, requiring rapid transitions between techniques.

While endotracheal intubation provides reliable ventilation, it may impede surgical access and is associated with airway trauma, swelling, bleeding, and irritation. This has increased interest in supraglottic strategies, particularly the laryngeal mask airway (LMA), which has been presented as a viable alternative in selected tracheal resections to reduce airway manipulation, tube-related stress on the anastomosis, and potentially improve postoperative airway symptoms [4]. However, neither LMA nor high-frequency jet ventilation (HFJV) is fail-safe. LMA efficacy depends on fit, does not address obstruction distal to the device, and does not permit lung isolation. HFJV, despite improving surgical exposure, can complicate CO₂ control and carries the risk of air trapping and barotrauma in patients with impaired exhalation [3]. Non-intubated tracheal surgery with spontaneous ventilation has also been reported in highly selected patients but requires substantial team experience and readiness to manage complications [5]. When airway-based strategies cannot reliably ensure oxygenation and CO₂ clearance in severe central airway obstruction, extracorporeal support becomes a rational rescue or planned adjunct. Extracorporeal membrane oxygenation (ECMO) has been used successfully in this setting and should be considered when the expected benefit outweighs the associated risks and resource demands [6].

Our patient had predictors of a difficult airway and later required lateral decubitus positioning with a chin-to-chest suture. While chin suturing protects the anastomosis, it also constrains airway manipulation once applied, underscoring the importance of early airway stabilization, continuous bronchoscopy reassessment, and a pre-agreed escalation ladder. In addition, thoracic surgical manipulation and mediastinal mobilization can dynamically alter airway and chest geometry, predisposing to malposition or loss of function of lung-isolation devices despite initially correct placement. Loss of lung isolation despite sequential bronchial blocker techniques highlights the dynamic nature of intraoperative airway conditions in tracheal surgery and aligns with reports that the primary strategy may fail despite correct initial placement, necessitating immediate alternatives [2,3]. Consistent with this, comparative clinical data show bronchial blockers require more intraoperative repositioning than double-lumen tubes, with repeated repositioning most frequently with EZ-Blockers, likely related to device design that limits how far the endobronchial limbs can be advanced, making them more vulnerable to displacement during mediastinal manipulation [7].

When lung isolation could not be restored, ventilation was transitioned to HFJV with intermittent apnea and, when feasible, cross-field ventilation. HFJV is often used to improve surgical exposure. As a rescue technique in shared-airway surgery, it has recognized pitfalls: difficulty maintaining partial pressure of carbon dioxide (PaCO₂) targets, the need for close monitoring, and the risk of air trapping or barotrauma in patients with impaired exhalation, including those with COPD [1,2,3]. In this case, frequent high-pressure alarms occurred, and oxygenation became increasingly limited after resection as blood and secretions contaminated both lungs, necessitating frequent bronchial toileting, performed either via suction catheter through the endotracheal tube or directly by the surgical team through the open tracheal field following resection. This reinforces that rescue strategies may become less reliable across surgical phases. HFJV was further limited by a mechanical and positional factor: the stiff jet catheter tip repeatedly abutted the inferior wall of the left mainstem bronchus, causing partial obstruction, elevated airway pressures (high-pressure alarms), and ineffective gas delivery. The jet catheter system is designed to mitigate this via a centering basket, with the main jet lumen exiting at the distal flexible tip. The centering assembly helps maintain a central position during insertion. In our case, the centering basket was not used because it was not available. Based on this experience, we recommend ensuring the availability and routine use of the centering basket for future cases requiring HFJV.

A further challenge was endotracheal cuff rupture with a major leak during ongoing airway manipulation. Beyond selecting among major airway and ventilation strategies, successful management also depends on recognizing and addressing secondary failures that threaten ventilation. Continuous cuff inflation using an auxiliary oxygen source provided a pragmatic salvage maneuver that reduced the leak and enabled safe completion; although optimal monitoring conditions were not feasible during this critical period, we ensured a persistent leak was present - evidenced by reduced tidal volumes and flow-volume loop changes - and communicated this to the surgeon to minimize the risk of airway fire. Although extracorporeal support (e.g., ECMO or cardiopulmonary bypass (CPB)) was not pursued given the absence of critical central airway obstruction or cardiovascular invasion, it remains an important consideration when airway-based strategies cannot ensure adequate oxygenation and CO₂ clearance; however, its significant resource demands and potential complications support reserving its use for cases where the anticipated benefits clearly outweigh the risks.

## Conclusions

This case highlights the need for a staged, multidisciplinary airway plan for tracheal surgery led by experienced subspecialty anesthetists, anticipating difficult airway constraints from positioning and chin-to-chest suturing and maintaining readiness and flexibility to transition between lung isolation, jet ventilation, intermittent apnea, and cross-field ventilation as conditions evolve. It also underscores the importance of being prepared to deploy simple salvage measures when needed.
